# The complete chloroplast genome and phylogenetic analysis of *Gentiana arethusae* Burkill (Gentianaceae) from China

**DOI:** 10.1080/23802359.2021.1984334

**Published:** 2021-10-05

**Authors:** Sisi Li, Wenbin Yuan, Shuilian He, Weiwei Bai, Hongzhi Wu

**Affiliations:** a College of Horticulture and Landscape, Yunnan Agricultural University, Kunming, Yunnan, China; b Department of Pratacultural Science and Technology, Yunnan Agricultural University, Kunming, Yunnan, China

**Keywords:** *Gentiana arethusae*, chloroplast genome, genome sequence

## Abstract

*Gentiana arethusae* Burkill is a perennial herb classified in the Gentianaceae. In this study, the complete chloroplast genome of *G. arethusae* was sequenced and analyzed. The chloroplast genome is 137,458 bp in length and encodes a total of 116 genes, including 71 protein-coding, 37 tRNA, and eight rRNA genes. The genome has a low GC content of 38.0%. Phylogenetic analysis of the genome of *G. arethusae* resolved it in a clade with *Gentiana obconica* and *Gentiana veitchiorum.* The complete chloroplast genome of *G. arethusae* will be helpful to study the genetic diversity and phylogenetics of the Gentianaceae.

*Gentiana arethusae* Burkill is a perennial herb with a high ornamental value, classified in the Gentianaceae. The species is mainly distributed in Yunnan, Hubei, Sichuan, and Tibet provinces in China (Flora of China Editorial Committee 1988). *Gentiana arethusae* generally grows on hillsides and grasslands at an altitude of 2000–3000 m (Flora of China Editorial Committee 1988). Many species of the genus can be used in Chinese medicine, including *G. arethusae* which is used to treat *rheumatic arthritis* (Qing et al. 2014). It is well-established that the chloroplast (cp) genome provides effective information for the identification, phylogenetic relationships, and population genetic analysis of plant species (Shang et al. 2020). In previous research, the evolutionary history and phylogenetic relationship of *G. arethusae* were generally studied using ITS and TrnL-F sequences (Favre et al. 2014, 2016), however, the complete cp genome of *G. arethusae* has not yet been deciphered. Here, the cp genome of *G. arethusae* was sequenced, assembled and analyzed, and will be contributed to the conservation, genetic improvement, and sustainable management of this species.

A wild individual of *G. arethusae* was sampled from Wantan town (110°23′E, 28°56′N, 2020 m), Wufeng County in Hubei province of China. The voucher specimen was deposited at the Herbarium of Yunnan Agricultural University (Contact person: Hongzhi Wu, hwu1128@163.com, No. 2021CD001). Total genomic DNA was extracted using a modified CTAB method (Doyle and Doyle 1987). The genome sequence was performed using Illumina Novaseq PE150. The analysis yielded 3.61 Gb of raw data that was further trimmed and assembled using SPAdes (Bankevich et al. 2012). The annotation of the cp genome was conducted with Geneious 8.0.2 (Kearse et al. 2012). The annotated genomic sequence was submitted to GenBank under accession number MZ603883.

The complete cp genome of *G. arethusae* is 137,458 bp in length and consists of two inverted repeats 23,865 bp, separated by a large single-copy region (LSC 77,907 bp) and a small single-copy region (SSC 11,822 bp). The overall GC content is 38.0%, with the GC content of the LSC at 35.6%, and the GC content of the SSC at 30.3%. The cp genome contains a total of 116 functional genes, including 71 protein-coding, 37 tRNA, and eight rRNA genes. Among them, 12 distinct genes (*trnK-UUU*, *trnG-UCC*, *trnL-UAA*, *trnV-UAC*, *trnI-GAU*, *trnA-UGC*, *atpF*, *rpoC1*, *petB*, *petD*, *rpl16*, and *rpl2*) contain one intron and three genes (*rps12*, *ycf3*, and *clpP*) contain two introns. All the rRNA genes in the genome sequence were located in the repeat region. Furthermore, the cp genome of *G. arethusae* was similar in gene structure and arrangement to the previously published *Gentian* species (Sun et al. 2018).

We used the complete cp genomes of *G. arethusae* and 29 other species from the genus *Gentiana* to reconstruct the phylogenetic tree designating S*wertia mussotii* and *Swertia hispidicalyx* as the outgroups. The maximum-likelihood (ML) analysis using RAxML 8.0 software (Stamatakis 2014) and the GTR + T nucleotide substitution model with 1000 bootstrap replicates resolved *G. arethusae* in a clade with *Gentiana veitchiorum* and *G. obconica* with high bootstrap values (Figure 1). Previous researchers used a combination of nuclear (ITS) and plastid (trnL-F and atpB-rbcL) markers to study the phylogenetic relationship of Gentianinae, the results showed that *G. arethusae* was closely related to *Gentiana lawrencei* and *Gentiana purdomiii*, while *G. veitchiorum* was relatively distant (Favre et al. 2014). However, in our research, the phylogenetic relationship between *G. arethusae* and *G. lawrencei* is rather distant (the complete cp genome of *G. purdomiii* has not yet been deciphered), and more close to *G. veitchiorum* (Figure 1). Some studies have shown that different conclusions are reached based on different molecular evidence for the same group (Zhang and Li 2011). The cp genome contains more nucleotide and amino acid information than gene sequences, and cp genomes are sequenced and analyzed more quickly and easily (Zhang and Li 2011). Therefore, the complete cp genome of *G. arethusae* will be helpful to determine the phylogenetic and evolutionary history of *Gentiana*.

**Figure 1.**
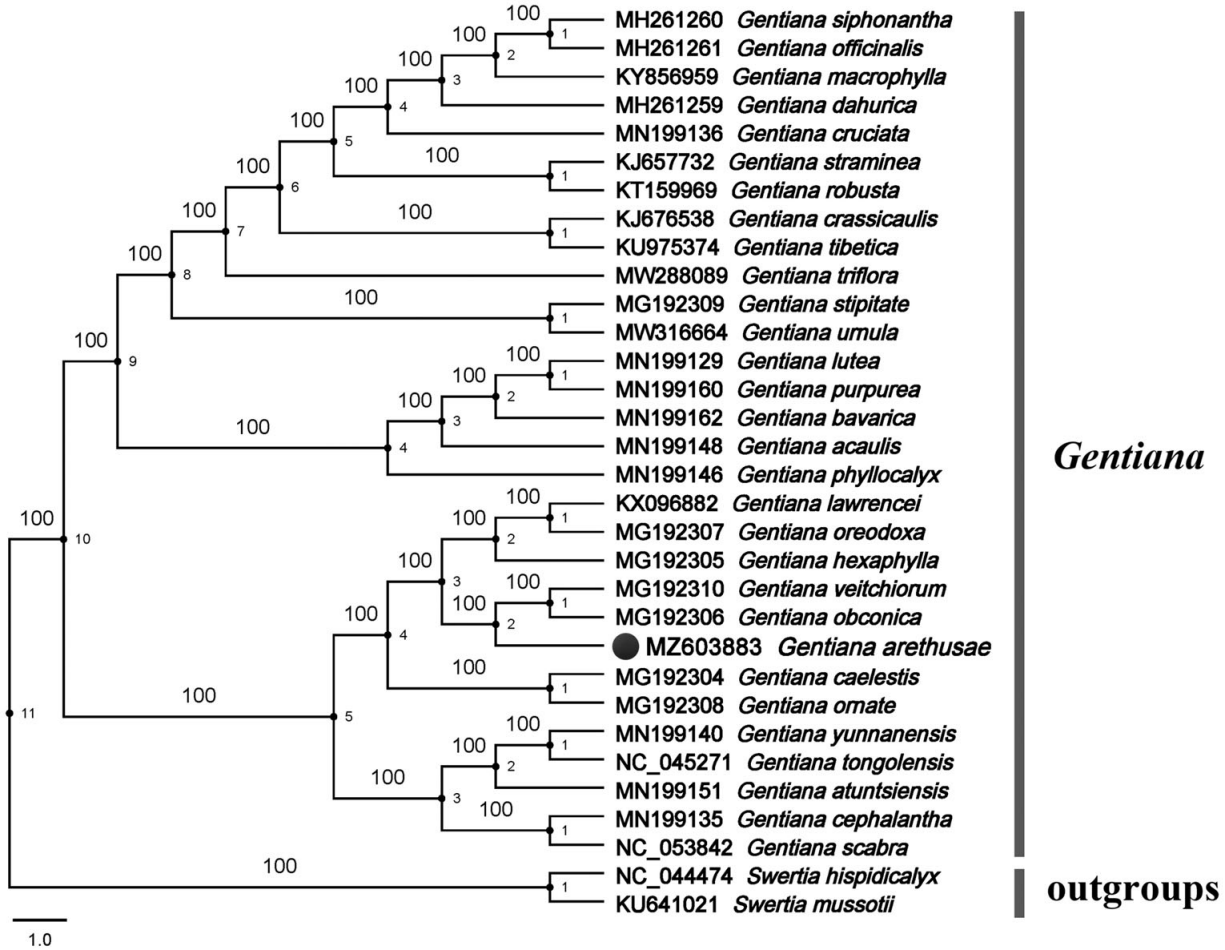
An ML phylogenetic tree of Gentianaceae based on the complete chloroplast genomes of 29 ingroup species and two outgroup taxa. Numbers at the nodes represent bootstrap support based on 1000 replicates.

## Data Availability

The data that support the findings of this study are openly available in GenBank of NCBI at https://www.ncbi.nlm.nih.gov/, reference number MZ603883. The associated BioProject, SRA, and Bio-Sample numbers are PRJNA749867, SRR15256470, and SAMN20423158, respectively.
